# Monitoring Beta-Blocker Therapy in Adolescents with Exercise-Induced Intraventricular Gradients Using Exercise Stress Echocardiography

**DOI:** 10.3390/biomedicines13082035

**Published:** 2025-08-21

**Authors:** Nuno Cotrim, Hugo M. Café, Jorge Guardado, Pedro Cordeiro, Rui Martins, Hortense Cotrim, Carlos Cotrim

**Affiliations:** 1 Hospital Distrital de Santarém, Avenida Bernardo Santareno, 3737B, 2005-177 Santarém, Portugal; nuno_cotrim1@hotmail.com; 2 Hospital Particular do Algarve, Urbanização Casal de Gambelas, Lote 2, Gambelas, 8005-226 Faro, Portugal; hmcafe@gmail.com (H.M.C.); pmcordeiro@hotmail.com (P.C.); 3 Unidade Cardiovascular (UCARDIO) Largo da Igreja Velha—Edifício CCA, Loja 1, 2350-325 Riachos, Portugal; jobeguardado@gmail.com; 4 Centro de Estatística e Aplicações (CEAUL), Faculdade de Ciências da Universidade de Lisboa Bloco C6, Piso 4, Sala 6.4.09, 1749-016 Lisboa, Portugal; ruimartins@ymail.com; 5 Universidade Católica Portuguesa (Sede—Campus de Palma de Cima), 1649-023 Lisboa, Portugal; hortensecotrim@gmail.com; 6 Heart Center, Hospital da Cruz Vermelha Portuguesa, Rua Duarte Galvão, 54, 1549-008 Lisboa, Portugal

**Keywords:** exercise stress echocardiography, intraventricular pressure gradients, lung comets, ß-blockers, adolescents, pediatric cardiology

## Abstract

**Background:** Treadmill exercise stress echocardiography (ESE) is both feasible and safe in the pediatric population. Although regional wall motion abnormalities (RWMAs) have limited diagnostic utility, Doppler studies frequently demonstrate significant intraventricular pressure gradients (IVPGs) during exercise. These IVPGs, which were observed in 39% of 258 previously studied adolescents, are absent at rest. Their detection provides valuable insight into exercise-related symptoms and abnormal findings on resting or stress electrocardiograms (ECGs). **Purpose:** To evaluate the effect of β-blocker therapy on the occurrence of intraventricular pressure gradients (IVPGs) in adolescents presenting with symptoms or abnormal findings on resting or stress electrocardiograms (ECGs). **Methods:** Exercise stress echocardiography (ESE) was repeated in 66 of 101 adolescents who were found to have developed intraventricular pressure gradients (IVPGs) during the initial assessment. All participants had normal resting echocardiograms, and all underwent follow-up evaluation while receiving β-blocker therapy. The study cohort included 15 females (23%) and the mean age of participants was 14.6 ± 1.7 years (range: 11–17 years). Comprehensive two-dimensional and Doppler echocardiographic assessments were performed at baseline and during β-blocker treatment. **Results:** During the initial ESE, the mean intraventricular pressure gradient (IVPG) was 105 ± 38 mmHg. Under β-blocker therapy, 37 adolescents no longer developed IVPGs while, in the remaining 29 adolescents, the IVPG was significantly reduced to a mean of 58 ± 32 mmHg (*p* < 0.0001). The mean heart rate at peak exercise decreased from 178 ± 15 bpm at baseline to 157 ± 9 bpm during the repeat ESE under β-blocker treatment (*p* < 0.0001). Clinical symptoms were reproduced in forty-seven adolescents during the initial ESE, but occurred in only seven adolescents during treatment (*p* < 0.0001). **Conclusions:** In adolescents presenting with symptoms or abnormal resting or stress ECG findings, and exertional intraventricular pressure gradients (IVPGs), oral β-blocker therapy either prevented the occurrence of IVPGs or significantly reduced their severity. These hemodynamic improvements were associated with the resolution of clinical symptoms in 85% of the symptomatic cohort.

## 1. Introduction and Aims

Exercise stress echocardiography (ESE) has revealed intraventricular pressure gradients (IVPGs) in many adolescents with exertional symptoms [1]. However, the clinical implications remain unclear. As IVPGs are linked to hyperdynamic left ventricular responses [2,3], β-blockers may offer therapeutic value. β-blockers have been used as a strategy in adult populations, including those with Syndrome X [2,3]. At present, however, pediatric evidence guiding such treatment is limited [4,5,6].

Although widely applied in adult cardiology, ESE remains underutilized in pediatrics, possibly due to concerns about its safety, the required learning curve, and the limited attention paid to ESE in the literature [7,8,9,10,11,12,13,14]. In adolescents with chest pain, conventional tests—ECG, Holter, stress testing—often yield inconclusive results [1]. Both ESE and MRI provide radiation-free imaging. ESE offers real-time assessment during physical exertion, whereas MRI—while offering superior tissue characterization—remains less practical for dynamic, exercise-based evaluations. Compared to ionizing imaging (CT, scintigraphy), ESE poses no radiation risk—a vital consideration given the cumulative exposure faced by adolescents with congenital heart disease [1].

**Figure 1:**
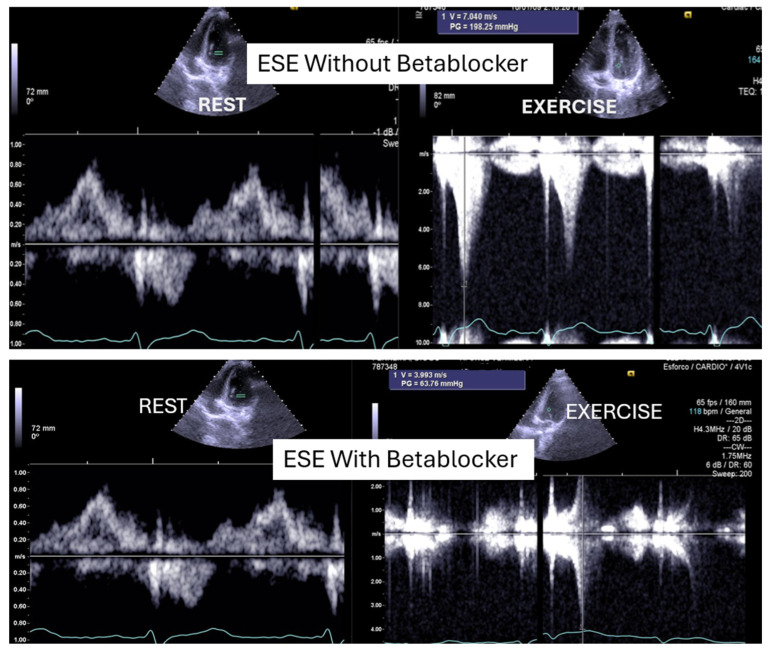
This figure shows an intraventricular gradient during exercise in an adolescent experiencing exercise-related chest pain and syncope, accompanied by elevated troponin levels (**top**). A significant reduction in the gradient is observed under beta-blocker therapy (**bottom**).

**Figure 2:**
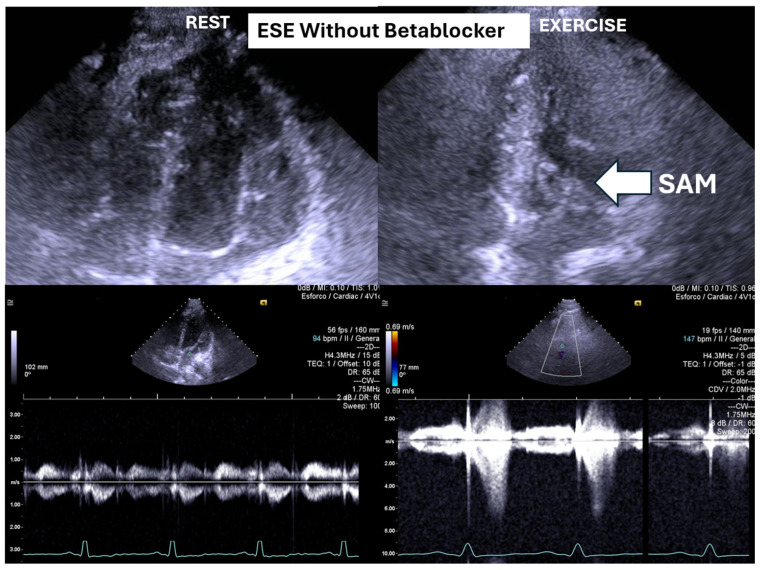
This figure depicts an intraventricular pressure gradient observed during exercise in an adolescent presenting severe exertional dyspnea, accompanied by pronounced systolic anterior motion (SAM) of the mitral valve.

ESE’s potential extends beyond diagnosis, as it may guide targeted therapy. Our experience supports its value in initiating β-blockers in selected pediatric cases (Figure 1 and Figure 2) [1,9].

Despite β-blockers being used for pediatric tachyarrhythmias [15], treatment regimens often rely on adult data [16,17]. In adults, β-blockers are standard for IVPGs, including those with hypertrophic cardiomyopathy [17], and preliminary findings suggest benefits in adolescents [15]. Ethically, pediatric care requires cautious use of radiation, favoring non-ionizing modalities and the lowest effective doses [1]. Adolescents’ limited autonomy demands physician-led decisions based on beneficence and considerations of long-term well-being [18,19,20]. Furthermore, excluding adolescents from trials has hindered the development of tailored therapies, perpetuating reliance on off-label treatments. The aim of this prospective, non-randomized study is to evaluate whether ESE can inform individualized β-blocker therapy in adolescents with exertional symptoms and IVPGs—with or without systolic anterior motion (SAM) of the mitral valve—detected during exercise.

## 2. Methods

In this study, a team of highly experienced adult cardiologists, who had performed an average of 500 exercise stress echocardiograms annually over the previous 20 years (totaling 1887 exercise stress echocardiograms in 2024), conducted exercise stress echocardiography on a group of adolescents, both with and without beta-blocker therapy. The treadmill and echocardiography equipment used were those available at the centers where the adolescents were evaluated between 2002 and 2019, and these varied over the years (including echocardiography systems from Philips^®^: Philiphs SONOS 5500, Koninklijke Philips N.V. Amsterdam, Netherlands, GE^®^: Vivid I, GE HealthCare Technologies, Inc, Heller International Building, Chicago, IL, United States. and Siemens^®^ Siemens SC2000 Siemens Medical Solutions USA, Inc., under the ACUSON SC2000 product line. 1230 Shorebird Way, Mountain View, CA, United States of America. However, the methodology, including the use of the Bruce Protocol previously reported in [1], remained consistent throughout the study period.

### 2.1. Sample

We performed stress echocardiography on a group of 66 adolescents, all of whom engaged in sports, at least at school. A total of 15 adolescents (23%) were female, and the mean age of individuals was 14.6 ± 1.0 years (ranging from 11 to 17 years). These adolescents all developed a significant intraventricular gradient (greater than 30 mmHg) without beta-blocker therapy, and all underwent ESE again after being treated with beta-blockers. The 66 adolescents were part of a larger cohort of 101 adolescents who developed IVPG during ESE [1], received beta-blocker treatment prescribed by their physicians, and underwent repeated ESE, as requested by their doctors, in our department between 2002 and 2019.

These adolescents did not have congenital heart disease, hypertrophic cardiomyopathy, or any abnormal findings on their echocardiograms. Exclusion criteria included the presence of left ventricular (LV) hypertrophy, mitral valve prolapse, or any other structural heart conditions.

Some adolescents presented unexplained exercise-related symptoms (chest pain in 22 adolescents, abnormal fatigue in 6 adolescents, dizziness/syncope in 21 adolescents) and/or alterations in the ECG or exercise stress test (7 adolescents). The doctors treating the adolescents prescribed the beta-blockers after the result of the first exercise stress echocardiogram. The adolescents first underwent exercise stress echocardiography without beta-blockers and later repeated the test, for the purposes of the present study, while on beta-blocker therapy within the following year. The methodology used [1] included evaluating wall motion during treadmill exercise, along with pulsed and continuous wave Doppler assessments to detect IVPG, as well as color Doppler imaging. Mitral valve motion was also analyzed, particularly for systolic anterior motion (SAM).

In all cases, beta-blockers were taken at breakfast on the morning of the exam.

At the time of the ESE performed during therapy, the beta-blockers prescribed by the adolescents’ physicians were as follows: atenolol 50 mg for two adolescents, and bisoprolol for sixty-four adolescents (2.5 mg for fourteen, 10 mg for one, and 5 mg for forty-nine adolescents).

This study was approved by the Ethics Committee of the Heart Center at the Red Cross Hospital in Lisbon. Adolescents who underwent an ESE performed by our team at the Red Cross Hospital, UCARDIO, and at the Hospital Particular of the Algarve were identified through a review of the internal EchoLabs database. Medical records were examined to collect adolescent data, including demographics, clinical diagnoses, indications for ESE, and test results. Written informed consent was obtained from parents or guardians, along with written informed assent from all study participants [19].

In the initial years of the study, examinations were entirely recorded on video. In later years, recordings were partially captured using the DICOM format.

An intraventricular pressure gradient was considered significant if it exceeded 30 mmHg at the end of systole, either before, during, or immediately after exercise. The Doppler echocardiographic parameters represented averages of three measurements taken from consecutive high-quality recordings.

### 2.2. Exercise Stress Echocardiography

A comprehensive echocardiographic evaluation was performed, including measurements of the left ventricular (LV) outflow tract, the LV mass index, the relative wall thickness, and the LV end-diastolic volume at rest. The maximum flow velocity in the LV outflow tract was assessed using continuous-wave Doppler from an apical five-chamber view to calculate the intraventricular pressure gradient (IVPG) before, during, and after exercise, with all assessments conducted in the upright position. This methodological approach is particularly relevant, as most physical activities performed by adolescents in daily life occur in an upright posture, thus providing physiologically meaningful insights [1,2,3]. The occurrence of IVPG during exertion is a frequently observed phenomenon in symptomatic adolescents or those with ST/T wave abnormalities on resting ECG or a positive exercise stress test, especially when actively investigated [1,2,3]. The cases reported in this study are of particular interest because post-exercise recovery was also conducted in an upright position—closely mimicking real-life conditions—and the observed gradients were comparable to those seen in patients with hypertrophic cardiomyopathy. Notably, the exercise stress echocardiograms were completed in the presence of symptoms such as fatigue, dizziness, or chest pain.

### 2.3. Ergometric Parameters

During ESE, the following parameters were evaluated: test duration (in seconds), systolic blood pressure at rest and peak exercise, heart rate at rest and at peak exercise, peak double product, and the presence of ST segment changes—specifically, ST depression of 1 mm occurring 80 ms after the J point. Additionally, any symptoms experienced during the test which resembled those that initially led to the patient’s evaluation were documented.

### 2.4. Statistical Analysis

The results were presented as means ± standard deviation for continuous variables, and as frequencies and percentages for categorical variables. Comparisons between the two assessments were performed using paired *t*-tests when the assumptions of size and distribution were met; otherwise, Wilcoxon signed-rank tests were used. The McNemar test was used to assess changes in paired proportions, enabling the evaluation of differences in dichotomous outcomes before and after intervention. Some of our adolescent patients missed the IVG measurement under BB therapy. In these cases, we only considered those with two complete measurements, without and with BB therapy. The analyses were performed using SPSS (version 30). All statistical tests were two-tailed and used a type 1 error rate of 0.05.

## 3. Results

On the resting echocardiogram, all the exams were considered normal with and without beta-blockers. No wall motion abnormalities were detected in any of the exams, with or without beta-blockers. In the complete group, IVPG (Figure 1) at peak exercise on the first assessment was 105 ± 38 mmHg, with mitral valve SAM in 28 adolescents (Figure 2). Among the 29 adolescents treated with beta-blockers, IVPG was 58 ± 32 mmHg, *p* < 0.0001.

During the first examination, 47 adolescents (71%) experienced the symptoms that had initially prompted the ESE. These symptoms included unexplained exercise-related issues, such as chest pain in twenty-two adolescents, abnormal fatigue in six adolescents, and dizziness/syncope in twenty-one adolescents, along with ECG abnormalities or exercise stress test alterations in seven adolescents. Under beta-blocker treatment, only seven adolescents (11%) reproduced the symptoms that had necessitated the initial ESE: chest pain in three adolescents, dizziness in three adolescents, and abnormal fatigue in one adolescent.

At the time of the second ESE, fifty-nine adolescents (89%) demonstrated clinical improvement and did not at that time experience the symptoms that led to their inclusion in the study. The main results of the variables studied are shown in Table 1.

**Figure 3:**
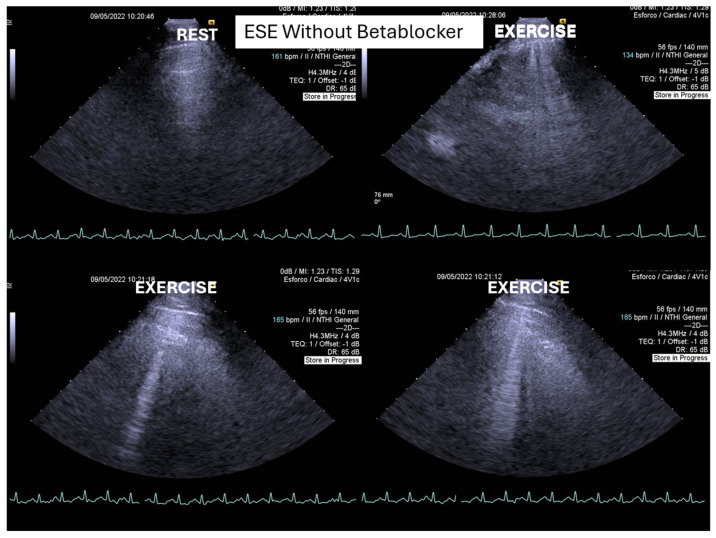
This figure depicts “lung comets” in the adolescent with the severe dyspnea shown in Figure 2 above.

One of these adolescents also developed “lung comets” [21,22,23,24,25]. These were investigated due to severe dyspnea associated with marked systolic anterior motion (SAM) and a significant intraventricular pressure gradient (IVPG) (Figure 3). To the best of our knowledge, this is the first report describing the presence of lung comets during exercise stress echocardiography (ESE) in pediatric patients, specifically in association with severe exercise-induced IVPG.

In patients with hypertrophic cardiomyopathy, the presence of lung comets has been associated with diastolic dysfunction, left ventricular outflow tract obstruction, and, in some cases, systolic dysfunction [21]. The evaluation of B-lines—both at rest and during stress—is increasingly recognized as a valuable tool for diagnosis of heart failure, assessment of its severity, monitoring of therapeutic efficacy, and enhancement of risk stratification. This clinical relevance has been underscored in recent cardiology guidelines and expert recommendations.

**Figure 4:**
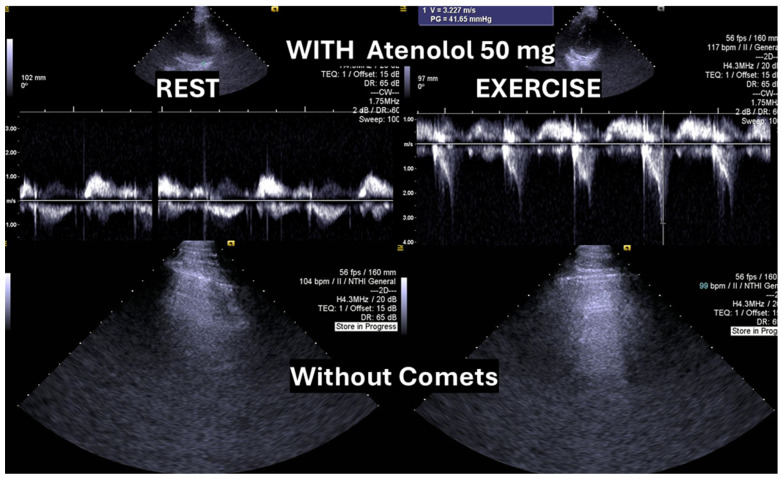
Absence of “lung comets” in the same adolescent following titration of beta-blocker therapy to atenolol 50 mg.

According to the 2021 universal definition of heart failure by the European Society of Cardiology, diagnosis is based on the presence of typical signs and symptoms, corroborated by elevated natriuretic peptide levels and/or objective evidence of cardiogenic pulmonary or systemic congestion [24]. In the present case, following a stepwise titration of beta-blocker therapy—from bisoprolol 2.5 mg, 5 mg, 7.5 mg, and 10 mg to atenolol 50 mg—the adolescent no longer exhibited lung comets (Figure 4).

**Figure 5:**
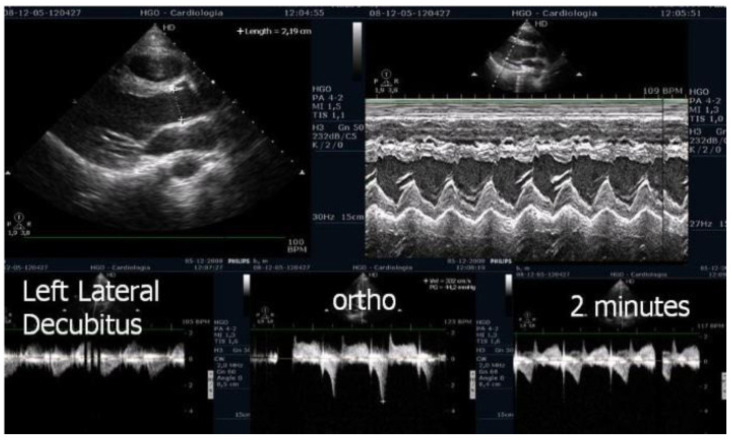
Echocardiography before exercise in a symptomatic adolescent in the left lateral decubitus position, and in the orthostatic position before and at the beginning of exercise [3].

**Figure 6:**
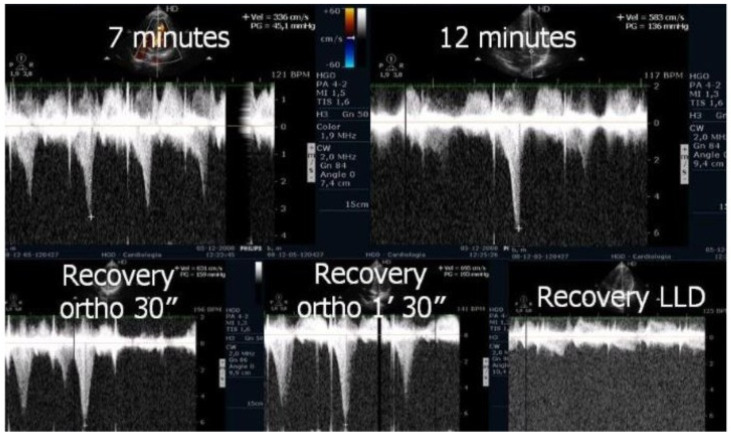
IVPG at different stages of exercise in the same symptomatic adolescent [3].

**Figure 7:**
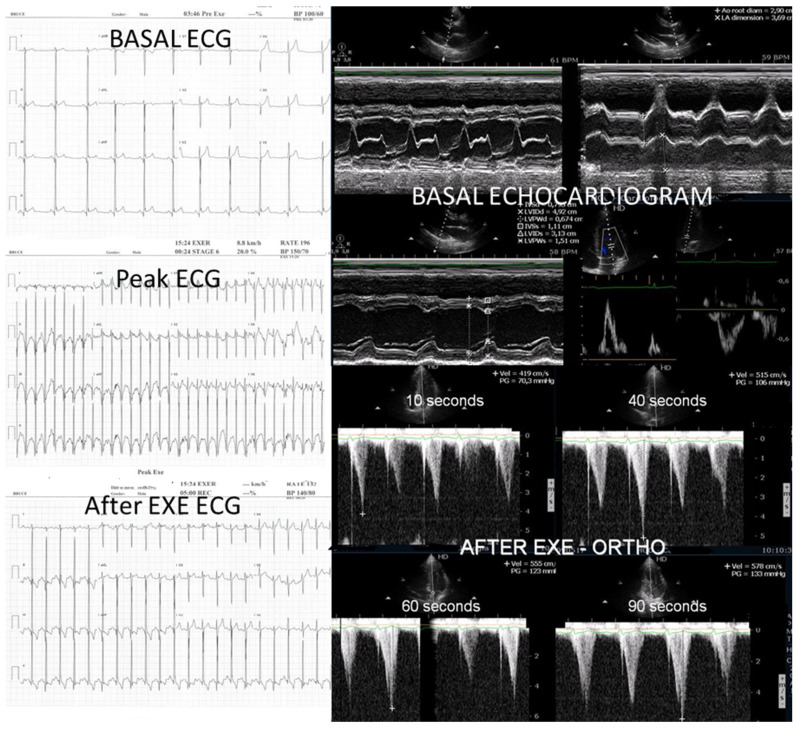
This composite image from an adolescent in the study with post-exercise angina shows ST alterations suggestive of ischemia occurring simultaneously with IVPG.

**Figure 8:**
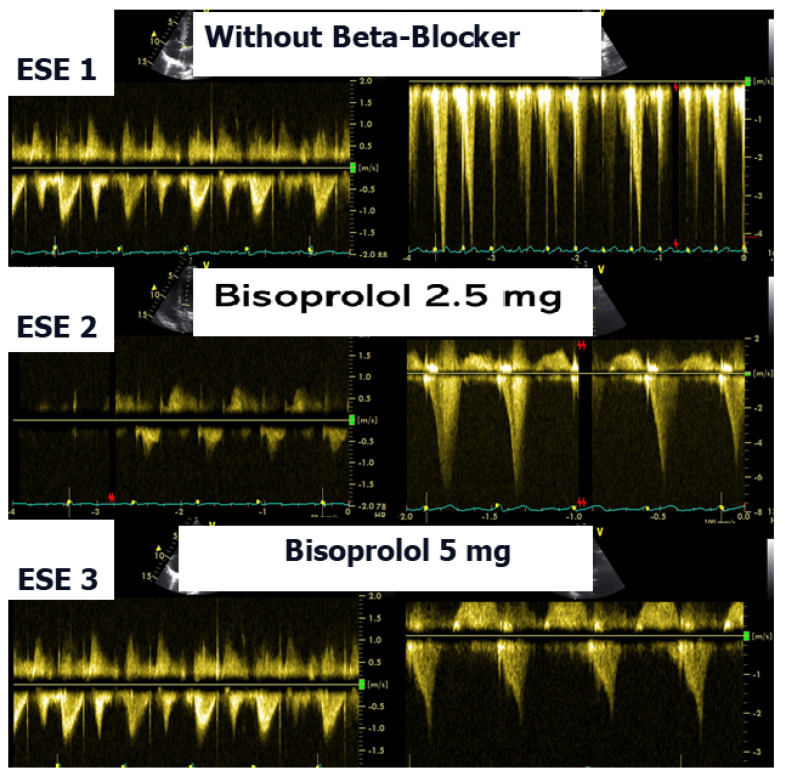
This composite image shows results obtained from an adolescent in the study during three exercise stress echocardiograms (ESE): the first shows significant IVPG; the second shows IVPG, despite 2.5 mg of bisoprolol having been taken; and the third shows nonsignificant IVPG, with 5 mg of bisoprolol having been taken.

We observed the development of intraventricular pressure gradients (IVPGs) in adolescents without left ventricular hypertrophy upon assumption of an upright posture prior to exercise—a phenomenon previously described in patients with hypertrophic cardiomyopathy [3]. At the onset of exercise (Figure 5), the IVPG initially decreased, likely due to increased preload resulting from activation of the lower limb musculature. However, as exercise progressed, the IVPG steadily increased (Figure 6). After exercise, maintaining an upright posture led to a more pronounced reduction in preload, compared to a supine position. This hemodynamic shift likely accounts for the post-exercise elevation in IVPG observed in most adolescents studied. This postural response may also explain the orthostatic recovery IVPG observed in one highly symptomatic adolescent who presented with exertional angina and ST-segment abnormalities (Figure 7). Additionally, Figure 8 illustrates the impact of beta-blocker therapy on ESE findings in one patient, including the titration of atenolol to a dose of 50 mg, underscoring the clinical relevance of individualized treatment.

## 4. Discussion

In our study, beta-blocker therapy was associated with reductions in heart rate, exercise-induced ST-segment alterations, systolic blood pressure, and, consequently, peak heart rate–systolic blood pressure product (HR × SBP). The incidence of intraventricular gradients (IVGs) (Figure 1) and systolic anterior motion (SAM) of the mitral valve (Figure 2) during exertion also decreased significantly. These hemodynamic improvements were accompanied by a notable reduction in symptoms during exercise testing and follow-up

Recent studies have confirmed that exercise-induced intraventricular gradients may occur in symptomatic individuals with structurally normal hearts, particularly during treadmill stress echocardiography, and may be associated with effort-related symptoms such as angina or dizziness [2,3].

The condition was managed using bisoprolol, which led to clinical improvements and substantial reductions in intraventricular pressure gradients (IVPGs). It has long been recognized that small intraventricular pressure gradients are a common phenomenon. Three mechanisms have been proposed to explain their significant increase during exercise: (1) an amplification of non-obstructive physiological gradients, (2) end-systolic obstruction due to mid-cavity obliteration of the ventricle, and (3) mid-systolic obstruction caused by systolic anterior motion (SAM) of the mitral valve, which restricts blood ejection [2,3].

However, SAM typically occurs when there is an alteration in the ventricular chamber geometry or the mitral valve apparatus. This was not the case with our adolescents. Nonetheless, studies have shown that intraventricular gradients can arise due to maneuvers that modify loading conditions in structurally normal hearts, such as those occurring during exercise [2,3]. Furthermore, dehydration during physical activity, reducing ventricular volume, may exacerbate the development of significant IVPGs and should be carefully monitored.

Subsequently, we identified the presence of intraventricular pressure gradients (IVPGs) in patients presenting with angina despite having angiographically normal coronary arteries. This observation prompted a study involving 91 patients diagnosed with cardiac Syndrome X, from which 20 individuals exhibiting IVPGs were selected for beta-blocker therapy. Treatment resulted in a significant improvement in clinical symptoms [2,3].

Treating pediatric patients, as demonstrated in this study, presents unique challenges, particularly because the use of beta-blockers in adolescents is often based on data extrapolated from adult populations. One major limitation is the absence of well-defined pediatric dosing guidelines during drug development. Historically, regulatory frameworks and pharmaceutical industry practices have largely excluded adolescents from clinical trials, resulting in a paucity of pediatric-specific pharmacological data. Nevertheless, existing evidence supports the clinical efficacy of beta-blockers in adolescents with left ventricular outflow tract obstruction [2,3]. Additionally, beta-blockers have been shown to alleviate symptoms potentially associated with exercise-induced intraventricular pressure gradients (IVPGs) in patients with and without hypertrophic cardiomyopathy [2,3,15,16,17]. Based on this body of evidence and ethical considerations, we elected to include beta-blocker therapy in our pediatric patient population.

In the present study, while symptom improvement was observed among participants, no significant change in functional capacity was detected. This finding aligns with current regulations, which generally do not prohibit beta-blocker use in most sports practiced by adolescents, including those practiced by athletes in this cohort [26,27]. The results also suggest that exercise stress echocardiography is a valuable tool for identifying symptomatic adolescents who develop intraventricular pressure gradients (IVPGs) during exertion and may particularly benefit from beta-blocker therapy.

### Study Limitations

One limitation of this study is its relatively small sample size. However, all 66 adolescents met strict inclusion criteria, as all were symptomatic and presented objective evidence of IVPG during exercise. As a result, they formed a carefully chosen group which represented those most likely to benefit from targeted beta-blocker therapy.

Another limitation is the absence of randomization and double-blinding for participants and investigators, which was not feasible due to ethical, logistical, and financial constraints. In our open-label design, the adolescents, their parents or guardians, and the investigators were all aware of the treatment being given. Additionally, the type and dosage of beta-blockers varied significantly, reflecting real-world treatment practices. All post-treatment tests were conducted during the second assessment, which may have affected certain results, such as treadmill test duration and adolescents’ symptom ratings. However, this would not have influenced objective measures like the presence and severity of IVPG, which remained the study’s primary focus. Further research, ideally through placebo-controlled randomized trials, is needed to assess the potential benefits of beta-blockers for such adolescents. The lack of recommendations for IVPG screening during exercise in adolescents with exercise-related symptoms or positive test results, combined with the absence of clear guidelines for beta-blocker treatment in these cases, highlights the need for additional studies.

## 5. Conclusions

In adolescents presenting with exercise-related symptoms, abnormalities detected during medical evaluations, such as in ECG or exercise stress tests, and an intraventricular pressure gradient (IVPG) identified during exercise stress echocardiography (ESE), oral β-blocker therapy proved highly beneficial. This treatment successfully prevented IVPG and systolic anterior motion (SAM), or significantly reduced their severity. These outcomes were linked to a marked reduction in peak exercise heart rate, and resulted in clinical improvement in 85% of the study participants (40 out of 47 adolescents) who exhibited symptoms during ESE.

Exercise echocardiography serves as an important diagnostic tool for assessing adolescents with ECG abnormalities, exercise ECG test irregularities, or exercise-related symptoms, even when a resting echocardiogram appears normal. It can aid in identifying potential candidates for beta-blocker therapy. The results of this study demonstrate the feasibility of customizing effective beta-blocker treatment based on exercise Doppler echocardiography findings, specifically by detecting intraventricular gradients and/or lung comets in symptomatic adolescents.

## 6. Potential Implications for Future Clinical Practice

Wider Integration of Exercise Stress Echocardiography in Pediatric Cardiology: Our findings support the broader implementation of ESE as a first-line diagnostic tool in adolescents presenting with exertional symptoms or abnormal electrocardiographic findings, even when resting echocardiograms are unremarkable. ESE enables the detection of dynamic cardiac abnormalities that may otherwise remain undiagnosed.

Personalized, Evidence-Based Beta-Blocker Therapy: ESE-guided detection of intraventricular pressure gradients (IVPGs) and systolic anterior motion (SAM) of the mitral valve allows for more targeted use of beta-blockers. This precision medicine approach has the potential to become standard care in managing exertional cardiac symptoms in pediatric populations.

Enhancing Pre-Participation Cardiovascular Screening Protocols in Adolescent Athletes: Current pre-participation evaluations for youth sports may benefit from incorporating dynamic testing modalities such as ESE. Identifying exercise-induced functional abnormalities can inform individualized management strategies and enhance safety among adolescent athletes.

Development of Clinical Guidelines for Pediatric IVPG and SAM Detection: The present study highlights a need for standardized diagnostic and therapeutic protocols addressing exercise-induced IVPG and SAM in adolescents, conditions that remain under-recognized in pediatric cardiology. Our results provide foundational evidence to support such guideline development.

Promotion of Multidisciplinary Collaboration: These findings underscore the importance of enhanced collaboration between pediatric cardiologists, general clinicians, and sports medicine specialists. An interdisciplinary approach is crucial to ensuring safe and effective care for physically active youths.

Need for Larger Randomized Controlled Trials (RCTs): Future research should include large-scale RCTs comparing clinical outcomes in adolescents receiving ESE-guided beta-blocker therapy versus untreated controls, or across different beta-blocker regimens. Such studies are essential to establish causal relationships and optimize treatment strategies.

## Figures and Tables

**Table 1:** Summary of variables assessed in the two exercise stress echocardiograms. Values are expressed as means ± standard deviation or as numbers (percentages), as appropriate.

Variable	ESE Without Beta-Blockers	ESE with Beta-Blockers	*p*-Value
Test duration (s)	695 ± 133	695 ± 117	0.786 (paired *t*-test)
Peak exercise HR (bpm)	184 ± 13	163 ± 25	<0.0001 (paired *t*-test)
Peak exercise SBP (mmHg)	152 ± 20	145 ± 20	<0.0001 (paired *t*-test)
Peak exercise DP (HR × SBP)	28,085 ± 5278	23,744 ± 3919	<0.0001 (paired *t*-test)
Peak IVPG (mmHg)	104.63 ± 38.14	57.83 ± 32.20	<0.0001 (Wilcoxon test)
Symptoms during SE (n, %)	47/66 (71%)	7/66 (11%)	<0.0001 (McNemar’s test)
SAM (n, %)	28/66 (42%)	11/66 (17%)	<0.0001 (McNemar’s test)
ST-segment alterations (n, %)	7/66 (11%)	3/66 (5%)	0.125 (McNemar’s test)

Abbreviations: DP—double product; HR—heart rate; IVPG—intraventricular pressure gradient; SAM—systolic anterior motion of the mitral valve; SBP—systolic blood pressure; ESE—exercise stress echocardiography.

## Data Availability

The data that support the findings of this study are available from the corresponding author upon reasonable request.
